# 

**Published:** 1905-03-15

**Authors:** 


### ANNOUNCEMENTS.

The Detroit Dental Society.—The annual clinic of this society was held February 11th, at the Detroit College of Medicine, and was more largely attended than any former meeting of the society. A large number of interesting and valuable clinics were given. In the evening a banquet was served at the Wayne Hotel which was attended by nearly one hundred and fifty guests. The toasts and entertainments made a delightful occasion. It does seem as though clinics and good cheer had their places in engendering professional *esprit de corps*.

—✦—

University of Michigan Alumni Clinic.—Friday and Saturday, February 17th and 18th, the alumni of the dental department inaugurated a new feature in the form of an annual clinic at the college. The attendance was large considering the fact that most of the railroads were snow-bound, and many who started for Ann Arbor had to turn back after coming a large part of the way. The clinic was held immediately after the close of the post graduate porcelain course, and naturally porcelain fillings and crown work constituted an important part of the program. Another interesting feature was a symposium on orthodontia, by Drs. M. T. Watson and V. H. Jackson. A large number of clinics on other subjects were given and the afternoon of Saturday was given up to clinics on surgery at the University of Michigan Hospital.

—✦—

Pennsylvania Examinations.—Examinations for registration in Pennsylvania will be held in Pittsburg and Philadelphia on the same days, June 6th to 9th, inclusive. Any one contemplating taking these examinations should write for papers and instructions to Dr. N. C. Schaeffer, Secretary Dental Council, Harrisburg, Pa.

—✦—

KANSAS DENTAL ASSOCIATION.—The 34th annual session will be held in Topeka, May 17th to 19th. Special attention will be given this year to the clinical program. A cordial invitation is extended to the profession. Headquarters will be at the Copeland Hotel,

F. O. HETRICK, *Secretary*,
Ottawa, Kansas.

—✦—

CENTRAL MICHIGAN DENTAL SOCIETY.—The next meeting of this society will be held at Lansing, March 28th and 29th. A cordial invitation is extended to all who can be present, as a good program is assured and some good clinics will be presented. A banquet will be a feature for Tuesday evening.

S. A. HORNING, *Secretary*,
Portland, Mich.

—✦—

MICHIGAN EXAMINATIONS.—The next examination for registration to practice in Michigan will be held at Ann Arbor, in the Dental College Building, beginning May 16th, 1905, at 9:00 A. M. All persons wishing to take this examination or register for practice in Michigan should write for particulars to C. H. Oakman, Secretary, Cleland Building, Detroit, Mich.

—✦—

NATIONAL ASSOCIATION OF DENTAL FACULTIES.—The annual meeting will be held at Buffalo, commencing a

2 P. M., on Thursday, July 27th, 1905. The executive committee will meet same day at 10 A. M. Special business to come before the National Association of Dental Faculties is the consideration of the proposed revision of the constitution and by-laws.

H. B. TILESTON, *Chairman Ex. Com.*,
JOHN I. HART, *Sec. Ex. Com.*

---

SOUTHWESTERN MICHIGAN DENTAL SOCIETY.—The next meeting of this society will be held at Kalamazoo, April 11th and 12th. A most cordial invitation is extended to all practitioners in that part of the State. The meeting will be a good one.

---

KENTUCKY STATE DENTAL SOCIETY.—The annual meeting of this society will be held May 15th and 16th, at Lexington, Ky. All ethical practitioners are urged to attend and assist in making the meeting a most valued one.

---

TRI-STATE MEETING.—As was stated in our January issue the Ohio State Society has declined to continue the Tri-State united effort to hold a dental convention every three years in either Ohio, Indiana or Michigan. The Michigan Society, whose turn it is to act as host this year, does not propose to submit to this decision and is laboring with the directors of the Ohio Society to get them to change their decision. If they should do so the meeting will be held in Detroit some time in July. Since this was written it has been decided that no Tri-State meeting shall be held this year.

American Dental Society of Europe.—The next meeting of this society will take place at Geneva, Switzerland, April 21st to 24th, 1905. A cordial invitation is extended to any American practitioners who may be in Europe this summer to participate in the proceedings of the society. Charles J. Monk, *Hon. Sec.*

---

The Lewis and Clark Dental Congress will be held in Portland, Oregon, July 17th, 18th, 19th and 20th, 1905. promises to be the largest ever held on the Pacific Coast.

The committee on clinics asks for voluntary clinics and table demonstrations from members of the profession and suggests that notice of the same be sent the committee as soon as possible.

In order that the program be complete, names of clinicians and clinics must reach the chairman not later than June 15th.

G. H. Nottage, *Chair. Com. on Clinics.*
Oregonian Bldg., Portland, Oregon.

---

New York Seventh District Society.—The annual meeting of this society will be held at Rochester, N. Y., March 28th and 29th, in the rooms of the Chamber of Commerce. The profession is cordially invited.

---

College Songs.—Hinds, Noble & Eldrige, publishers of New York City, have sent us a copy of "The Most Popular College Songs," for review. The selection is certainly well chosen and beautifully printed. It would be a pleasant reminder of old college days to have a copy in each dentist's home, and no more interesting book could be placed in the ibrary of his waiting room.

